# Case Report of a Chronic Wound Due to Venous Insufficiency Following a Traumatic Arteriovenous Fistula

**DOI:** 10.4236/ojcd.2021.112003

**Published:** 2021-06-29

**Authors:** Jialiang Chen, Louis Chebli

**Affiliations:** Department of Vascular Surgery, CHU Brugmann, Brussels, Belgium

**Keywords:** Arteriovenous Fistula, Chronic Wound, Trauma, Venous Insufficiency

## Abstract

Chronic lower limb wounds are common. They can be of arterial or venous origin. In this article, we will present a clinical case of a 30-year-old patient with a chronic injury to the right medial malleolus. In his history, we can note a gunshot wound to the right leg. Ultrasonography and CT angiography helped in the diagnosis of traumatic arteriovenous fistula. The patient underwent a fistula embolization which allowed the wound to heal. The clinical presentation, additional examinations and the latest treatment recommendations will be discussed in this article.

## 1. Introduction

Arteriovenous fistulas are abnormal connections between an artery and a vein. Whilst some malformations arteriovenous are congenital, most are post-traumatic or iatrogenic. This renders the diagnosis even less easy in peaceful countries because of their scarcity. Moreover, diagnosis is difficult because of the large spectrum of clinical manifestations and sometimes late. These are pain, varicosity, swelling of a limb, thrill or audible bruit locally, diminished pulses distally and the downstream pressure is reduced [1]. When the arteriovenous fistula is large, there is ischemia claudication, steal syndrome and cardiac decompensation [2]. Here, we present the case of a man who developed a chronic sore following venous insufficiency caused by an acquired traumatic arteriovenous fistula.

## 2. Case Report

**Figure 1.**
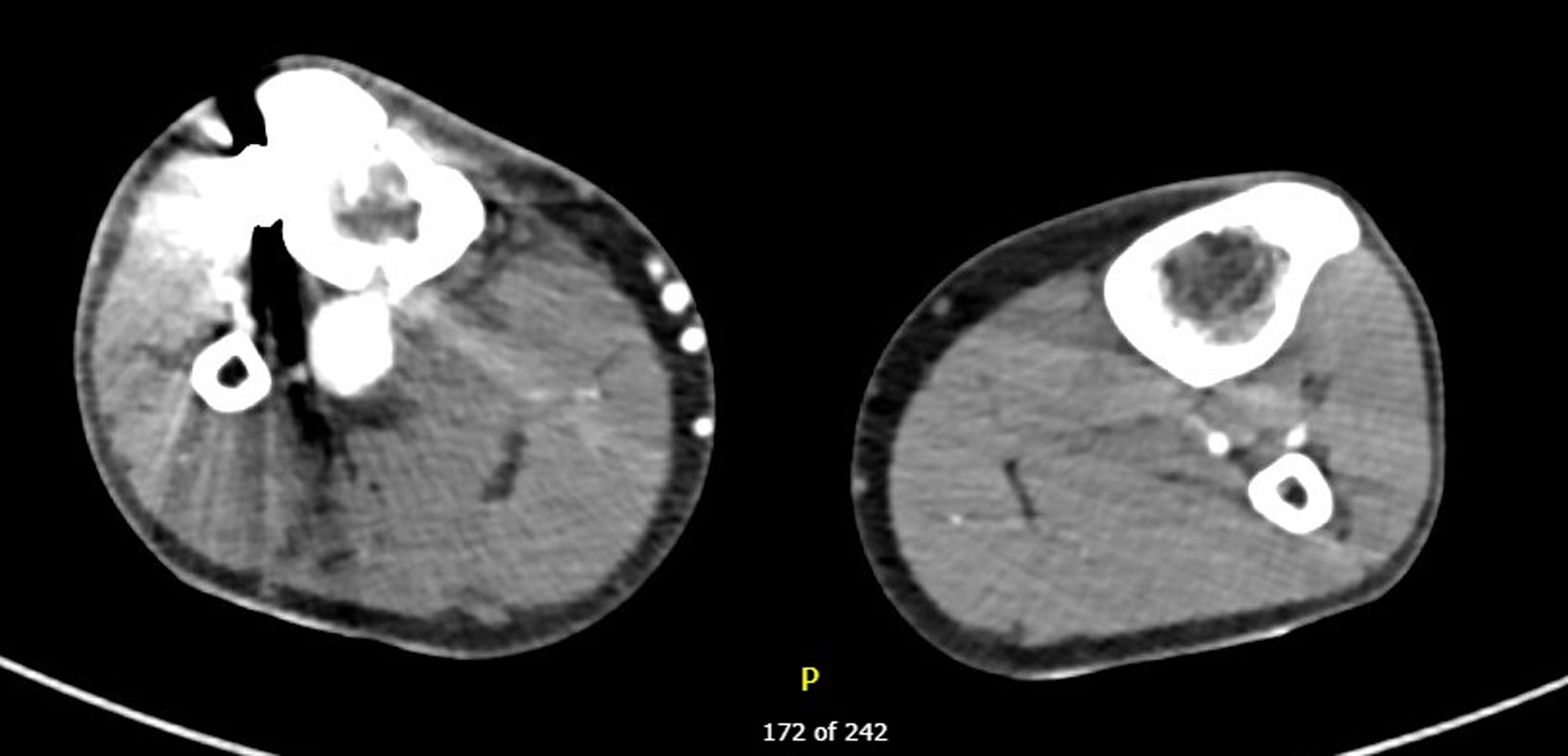
CT scanner in arterial phase where the contrast product passes into arteriovenous fistula.

**Figure 2.**
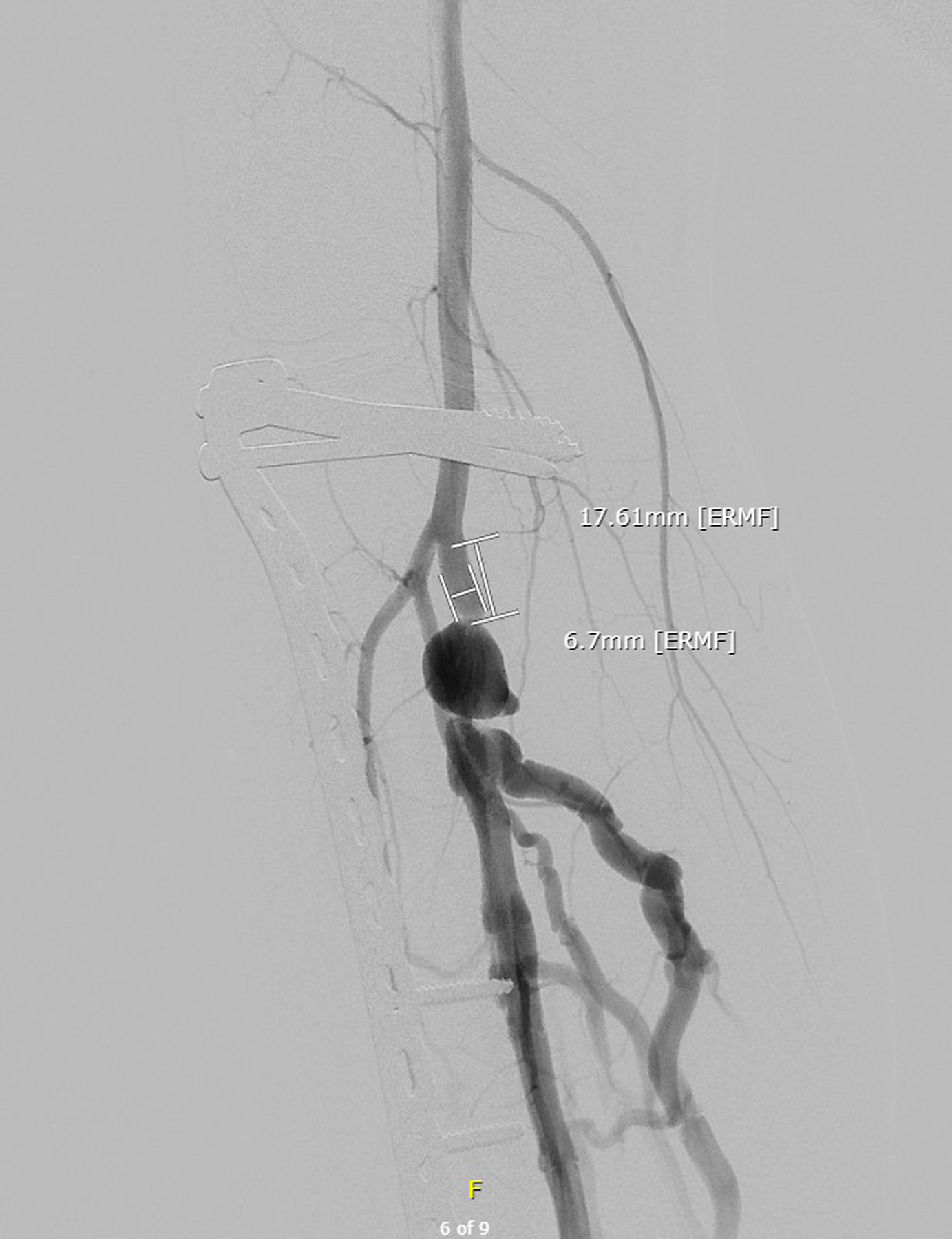
Arteriography of the arteriovenous fistula on the right tibial posterior artery.

This is the case of a 30 years old man. He presents with a chronic ulcer on the right internal malleolus. This lesion is very painful and productive. He has been trying local treatment for 2 years without any change. In his history, we note a bullet wound in his right leg in 2014 which was treated by external fixator, then an osteosynthesis by plate. In 2018, after an ultrasound proving there was a venous insufficiency of his right external saphenous vein, the patient benefitted from a right external saphenous vein crossectomy. After that, he developed headaches, leg swelling with pitting edema and no improvement in the wound. When we saw him for the first time, he continued to have significant pain despite first grade painkillers. We can notice a right internal malleolus wound, a swollen and indurated right leg. At first, we prescribed second level painkillers and we asked for an ultrasound for deep vein thrombosis. The ultrasound was normal. At the second consultation, we re-examined the patient and the wound is stable, but we palpated a thrill in the saphenous vein. This sign is present in the arteriovenous fistula. We requested a CT scan of the lower limbs (Figure 1). This confirms our hypothesis. There is a subarticular posterior tibial arteriovenous fistula that supplies the superficial and deep venous network of the right leg. Due to the presence of osteosynthesis material obstructing clear view of the arteriovenous fistula, we decided to perform an arteriography which confirms the arteriovenous fistula coming from the right posterior tibial artery (Figure 2).

**Figure 3.**
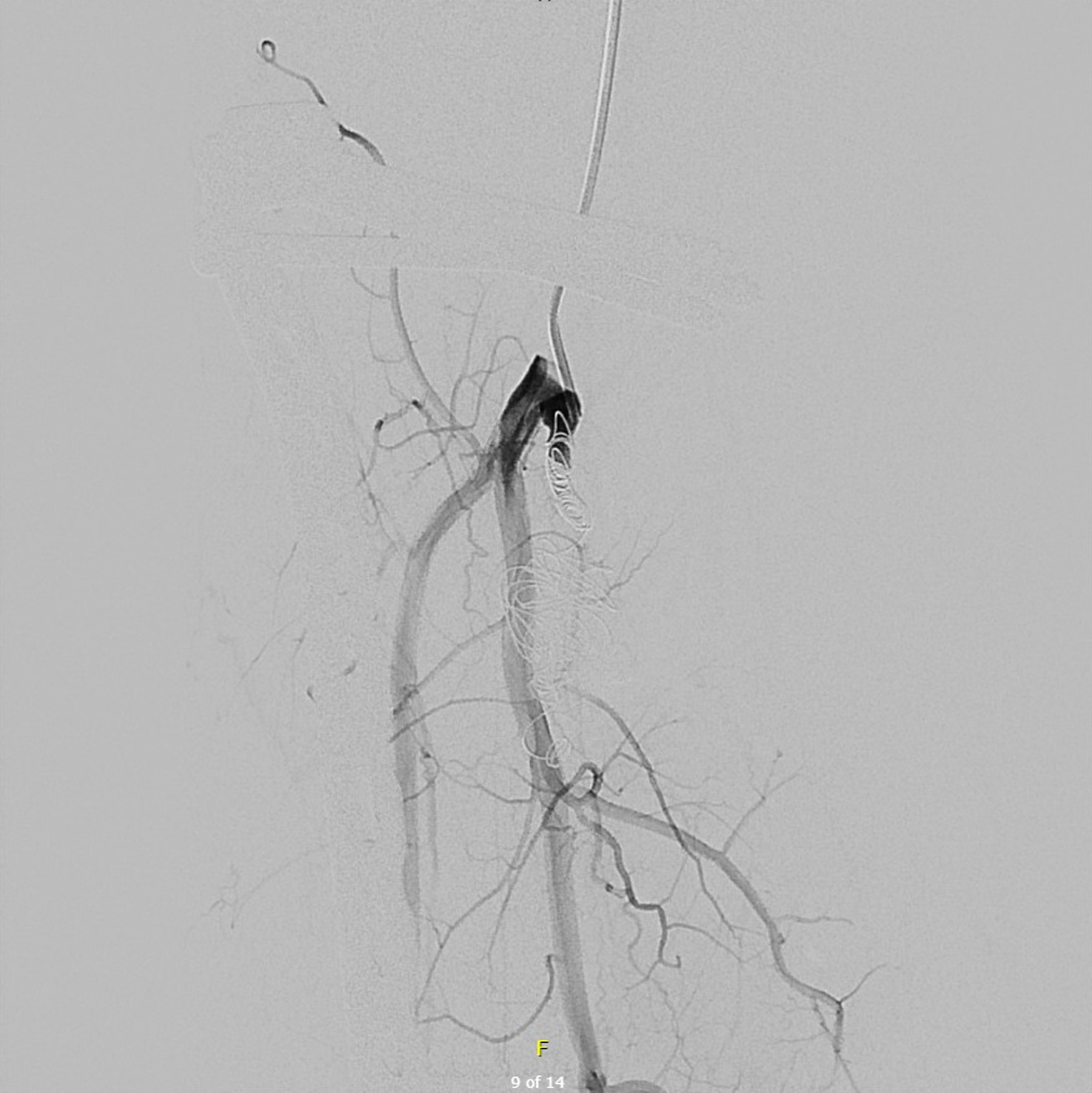
After placement of coil into arteriovenous fistula.

**Figure 4.**
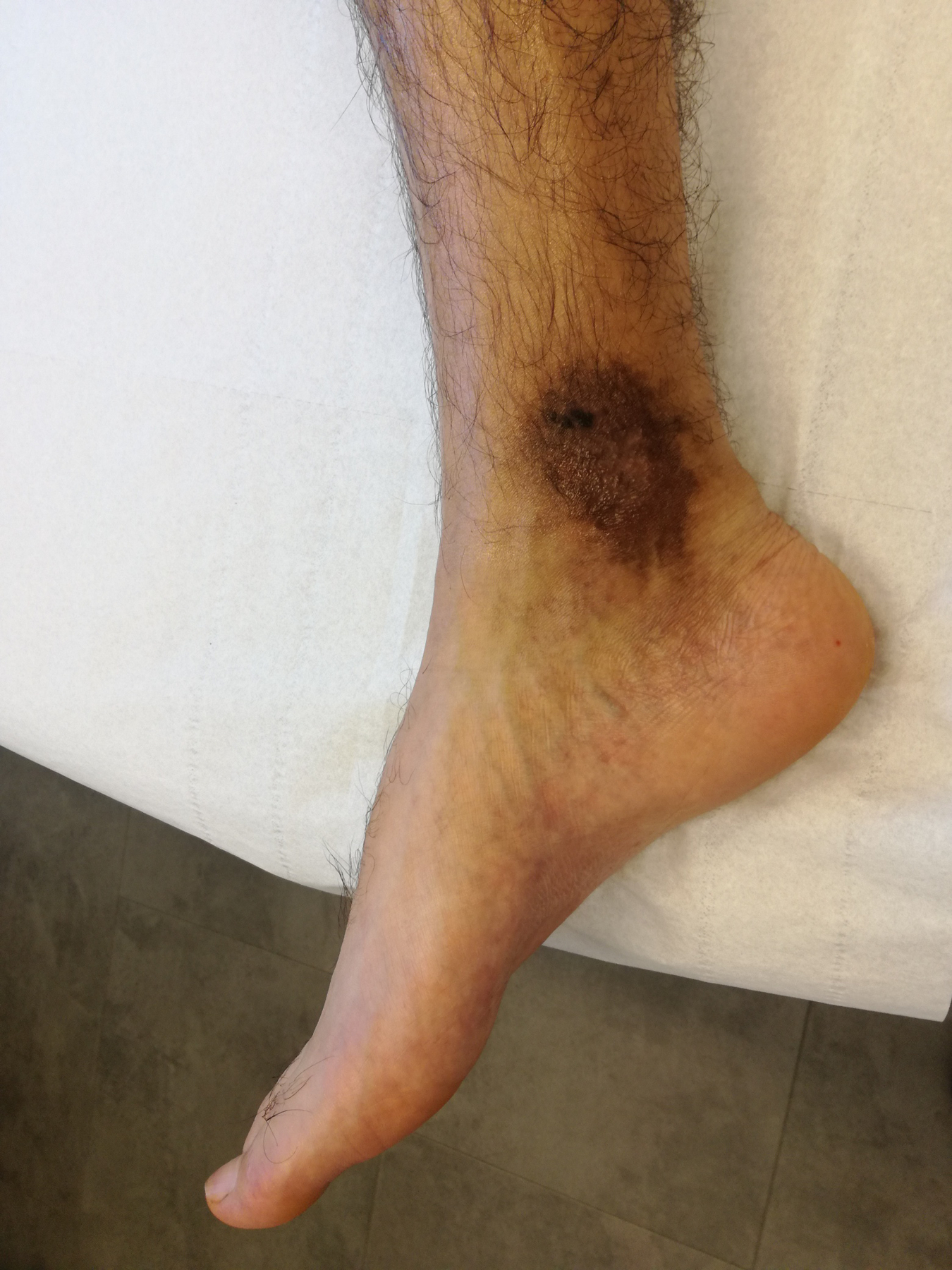
Result after one month.

Therefore, in view of the position of the fistula, it seemed reasonable to us to carry out and embolization. We decided to discuss this patient with the interventional radiologists because in Belgium, it is these doctors who perform the embolizations. We decided to embolize the right posterior tibial artery in this young patient. The patient arrives the morning of the operation and stays over-night for monitoring. If all goes well, he can leave the hospital. The procedure is done under local or general anesthesia depending on the patient’s condition and the patient’s wishes. We used Nester^®^ Embolization Coils 0.018 with an immediate satisfactory result (Figure 3). The patient was discharged without complications. At one month, at his visit to the outpatients department, we noted the start of a healing process of the ulcer (Figure 4). Thereafter, we continued to see him every three months until the wound healed completely.

## 3. Discussion

We live in an area where gunshot wounds are rare. As a result, we do not think directly of the consequences of these traumas. 2% - 3% of vascular trauma is complicated by arteriovenous fistula [2]. Moreover, clinical manifestations appear years later [3]. Patients often report a chronic injury. On clinical examination, there are signs of venous insufficiency: legs swelling, varicosities, pain on legs at the end of the day… However, there are the presence of a thrill, audible bruit, diminished distal pulse and the downstream pressure is reduced [1] [4] [5]. When the arteriovenous fistula is large, there are ischemia claudication, steal syndrome and cardiac decompensation [2].

The diagnosis is made with the help of the clinical examination, but also with an ultrasound which is however operator dependent. This examination sometimes makes it possible to visualize the arteriovenous fistula directly, but can especially show indirect signs such as a decrease in downstream arterial flow. In addition, the spectrum of the vein is arterialized [5]. However, neither the topography nor the exact shape of the arteriovenous fistula can be specified [6]. In our case, the radiologist ruled out a deep vein thrombosis, but did not describe the venous flow that could have directed us to the pathology. Note that in some hospitals, there is an ultrasound in the vascular surgery consultation room. This makes the diagnosis more quickly and to speed up the treatment. This is unfortunately not our case. CT angiography describes the location, shape and topography. In general, this examination is sufficient for the diagnosis and the therapeutic choice [3]. However, there are situations in which artefacts (orthopedic equipment, patient movements, etc.) can prevent proper visualization of the area of interest. Angiography helps determine the shape and location of the pathological area. However, it does not show the relationship with neighboring structures. However, it allows an endovascular procedure to be performed at the same time of operation. Nuclear magnetic resonance can measure flux, but this test is expensive and not always available in our regions. In addition, there is a contraindication when the patient has suffered a gunshot wound or the presence of metallic debris in the tissues.

Treatment for arteriovenous fistula can be surgical or endovascular. The advantage of the latter is shorter operating time, reduced risk of bleeding, less post-operative pain and fewer complications [1]. The length of hospital stay is also shorter. The technique consists of placing “stents”, “coils” or gel. However, there are limitations, such as the risk of a “stent” fracture in the vessels at the joints [7] [8]. Therefore, it is contraindicated to place them at these levels. In addition, at the level of complications, we can also note a risk of limb ischemia or pulmonary embolism.

Surgical treatment is recommended only when there is no possibility of endovascular treatment. It consists of ligating or resecting the arteriovenous fistula followed by veno-venous and arterio-arterial anastomosis. When the situation does not allow it, it is advisable to perform a venous or prosthetic bypass in order to avoid venous insufficiency. Note that the permeability of long-term venous by-passes is better than prostheses. We can draw an analogy with the treatments in dialysis patients who have had a defective arteriovenous fistula.

## 4. Conclusion

In the presence of a chronic wound with a history of trauma, the diagnosis of arteriovenous fistula should be considered. Ultrasound is a good additional diagnostic test, but not sufficient to determine the shape or topography before treatment. The CT angiogram seems to us to be the ideal choice to determine the location and the therapeutic choice. Endovascular treatment is preferred.
